# Children’s bond with companion animals and associations with psychosocial health: A systematic review

**DOI:** 10.3389/fpsyg.2023.1120000

**Published:** 2023-06-23

**Authors:** Daniëlle Groenewoud, Marie-Jose Enders-Slegers, Roeslan Leontjevas, Annemiek van Dijke, Tynke de Winkel, Karin Hediger

**Affiliations:** ^1^Faculty of Psychology, Open University, Heerlen, Netherlands; ^2^Department of Primary and Community Care, Radboud University Medical Centre, Radboud Institute for Health Sciences, Radboudumc Alzheimer Center, Nijmegen, Netherlands; ^3^Brijder the Hague, Parnassia Group, Amsterdam, Netherlands; ^4^NeLL/Leiden University Medical Centre, Leiden, Netherlands; ^5^Faculty of Psychology, University of Basel, Basel, Switzerland

**Keywords:** child, attachment, companion animal, human–animal bond, psychosocial health

## Abstract

**Background:**

Companion animals can fulfill children’s attachment needs. A secure attachment to humans is positively associated with psychosocial health, therefore, the extent to which this applies to a strong child-companion animal bond is worth examining.

**Aims:**

We aimed to gain insight into the current literature regarding the bond between children and companion animals and psychosocial health. Secondary, we also synthesized evidence about the (1) characteristics of children and companion animals and the strength of their bond; (2) the correlations between attachment to humans and the child-companion animal bond; and (3) the instruments used to measure the child-companion animal bond.

**Method:**

According to PRISMA guidelines, we searched three major electronic databases (PubMed, EBSCOhost, and Web of Science) in September 2021 and included records with the following criteria: peer reviewed English articles with quantitative and qualitative data on child-companion animal bonds and children’s psychosocial health. Reports with participants younger than 18 years of age with a family owned companion animal were included. Two authors performed the screening and determined eligibility according to a predefined coding protocol.

**Results:**

The search revealed 1,025 unique records, of which we included 29 studies. Some positive associations were reported between the strength of the child-companion animal bond and children’s psychosocial health outcomes like empathy, social support, and quality of life, although some results were contradictory. We found differences in associations between a child’s gender, companion animal species and the strength of the child-companion animal bond. A secure attachment style to parents was positively associated with a stronger child–companion animal bond. Most of the instruments currently used, measure the strength of the bond.

**Discussion:**

This review suggests that the child-companion animal bond could be beneficial for children’s psychosocial health, but some results were inconclusive. Also, not every relationship develops into an attachment. Since a strong bond with animals might not be the same as a secure attachment, we advise to modify human attachment instruments, in order to effectively study children’s attachment to companion animals. Lastly, research designs that are able to investigate the causality of the relationship between the child-companion animal bond and psychosocial health are required.

## 1. Introduction

The bond between humans and their companion animals is often described as attachment. Companion animals can fulfill the attachment needs of humans such as the need to be accepted, loved, recognized and appreciated (Kurdek, 2009; Julius et al., 2012; Zilcha-Mano et al., 2012; Mikulincer and Shaver, 2020). Many children consider companion animals as part of the family, therefore they can constitute attachment figures (Strand, 2004; Kurdek, 2009; Walsh, 2009; Drogt, 2018).

Attachment, which is formed and maintained by attachment behaviors, can be described as an enduring bond or tie between two individuals (Ainsworth et al., 1978; Ainsworth, 1989). For a bond or relationship to qualify as an attachment, the following specific criteria must be met: the relationship is of emotional importance and thus providing a secure base and safe haven, and distress upon separation and relief upon proximity seeking are evident (Bowlby, 1988; Ainsworth, 1989). Moreover, attachment can have different styles. An attachment style can either be secure or insecure avoidant, insecure anxious or insecure disorganized. About 60–65% of the general population in the Western world is securely attached (Van Ijzendoorn and Kroonenberg, 1988; Andreassen and West, 2007). The different attachment styles are often described along two dimensions: anxiety and avoidance (Bartholomew and Horowitz, 1991). Attachment anxiety refers to a person’s thoughts on the availability of the attachment figure and the need for proximity in times of stress. Attachment avoidance describes a persons’ (mis)trust in others and the preference of being self-sufficient rather than depending on others in times of need (Mikulincer and Shaver, 2003). Bowlby (1969) described that children form multiple attachments to parents, grandparents, siblings, partners, and friends, with all these individuals represented within a (mental) network as attachment figures.

The strong and enduring emotional bond that humans experience with companion animals has similarities with interpersonal relationships (Enders-Slegers, 2000; Kurdek, 2009; Zilcha-Mano et al., 2012). It has been shown that a bond with companion animals can fulfill the attachment criteria. Companion animals provide a secure base from which to explore, and provide a safe haven in times of stress (Kurdek, 2009; Zilcha-Mano et al., 2011; Julius et al., 2012; Meehan et al., 2017; Hoffmann et al., 2018). Humans not only seek closeness to a companion animal but they also enjoy their company (Enders-Slegers, 2000). Furthermore, separation from a companion animal often triggers anxiety and distress in individuals, and when a companion animal dies, feelings of grief and sadness are often experienced (Jarolmen, 1998; Hunt et al., 2008; Zilcha-Mano et al., 2011; Miller et al., 2014). Studies with adults have shown that secure and insecure attachment styles can be applied to human-companion animal attachment (Beck and Madresh, 2008; Zilcha-Mano et al., 2011, 2012). Nevertheless, not all relationships with companion animals can be classified as an attachment, as some meet only a few of the attachment criteria, Such bonds, that only partly fulfill the attachment criteria but still are able to fulfill attachment needs, are characterized as a companionship, support, affiliative bond, or ownership (Crawford et al., 2006; Rosenthal and Kobak, 2010). Moreover, when describing the human-animal bond, it is important to differentiate between an attachment, companionship, or mere social support from companion animals. To do this, instruments that differentiate between these forms of bonds are required. Although a broad variety of instruments have been developed and validated to determine the nature of a child’s bond with their companion animals (Poresky et al., 1987; Anderson, 2007), it is unclear if they can determine an attachment that fulfills the four attachment criteria, namely safe haven, secure base, proximity seeking and separation distress. Further knowledge regarding the instruments used to measure the scale of the child-companion animal bond could help differentiate between a genuine attachment, companionship, or social support by the companion animal.

The effects of attachment styles on children’s psychosocial health such as emotion regulation, anxiety, depression, social competence, empathy, prosocial behavior and externalizing behavior problems, has been studied widely (Fearon et al., 2010; Granqvist et al., 2017; Groh et al., 2017). A secure attachment style is associated with a positive sense of self-worth, empathy and the ability to regulate emotions and effective coping strategies (van Dijke and Ford, 2015; Cooke et al., 2019). An insecure attachment style is associated with internalizing symptoms such as anxiety, depression and a negative sense of self-worth, and externalizing symptoms such as difficulties to regulate emotions and behavioral problems (Brumariu and Kerns, 2010; Colonnesi et al., 2011). This can last throughout childhood into adulthood and potentially develop into mental disorders (Van Dijke and Ford, 2015).

However, all these results refer to human-human attachment. Research has shown that adults who develop an anxious attachment to a companion animal, when controlled for interpersonal attachment insecurity, can experience psychological distress (Zilcha-Mano et al., 2011). This could imply that the bond with companion animals is related to psychosocial health. However, there is still a lack of knowledge concerning children’s bond with companion animals and their psychosocial health. Evenmore, it is unclear if the bond between a child and companion animal is differentiated between attachment, companionship, or social support from a companion animal.

The extent to which a child’s bond with a companion animal is associated with their psychosocial health, is largely unknown. Although children with insecure attachments styles often distrust humans, the relationship they form with companion animals can be essentially different (Parish-Plass, 2008; Wedl et al., 2015; Kertes et al., 2017). For example, Melson and Fine (2015) stated that: “because companion animals are readily available and nonjudgmental, they can provide a feeling of support and compassion when humans are unavailable, unable or unwilling.” A comprehensive review study by Purewal et al. (2017) showed that companion animal ownership by children is associated with benefits such as a positive self-esteem, social competence, reduced stress and the development of empathy. They also state that the bond with a companion animal, or attachment to a companion animal could be an important factor in explaining the effects of owning a companion animal. It is therefore important to differentiate between an attachment, companionship, or mere social support from companion animals. In scientific literature about the human-animal bond, the terms ‘bond’ and ‘attachment’ are often used interchangeably. In this article, the term ‘bond’ is used when articles describe the strength of the child-companion animal bond. ‘Attachment’ refers to children’s attachment styles to others (humans and companion animals).

The primary aim of this systematic review is to summarize and evaluate the current knowledge regarding the correlation between a child’s psychosocial health and their bond with companion animals. We further synthesized evidence to highlight the specific characteristics of the children and their companion animals which are correlated with the strength of the children-companion animal bond. Additionally, we investigated the correlation between a child’s bond with a companion animal and their attachment to human attachment figures. Lastly, we presented an overview of the applied instruments which measure the strength of a child-companion animal bond.

## 2. Methods

### 2.1. Procedure and search strategy

The first author conducted a systematic literature search in electronic databases (PubMed, EBSCOhost, and Web of Science) in September 2021. The following search terms were used: “companion animals” OR “animals” OR “dogs” OR “cats” AND “children” OR “adolescents” OR “youth” OR “child” OR “teenager” AND “attachment” OR “attachment style” OR “social support” OR “attachment theory” OR “bond” OR “human animal bond.” If applicable, we performed the search by restricting the age to within the range of 0–18 years old, the language to English and Dutch (Pubmed and EBSCOhost) and peer reviewed articles only (EBSCOhost). Also, references from relevant articles were hand checked by the first author. All obtained records were imported into Rayyan software, where all duplicates were removed, and the screening was performed independently by the first and fifth author. Titles of the retrieved records were screened and irrelevant references were excluded. The abstracts and full texts of relevant publications were screened by the first and fifth author. Ambiguities were resolved by consensus between the two researchers involved in the screening process. Study identification, screening and eligibility determination was performed according to the Preferred Reporting Items for Systematic Reviews and Meta-analyses (PRISMA) guidelines (McInnes et al., 2018; Page et al., 2021). This systematic review was not registered at Prospero.

### 2.2. Eligibility criteria

We included records according to the following criteria: articles written in English or Dutch reporting quantitative and/or qualitative data about children’s psychosocial health and their bonds with, or attachment to companion animals; articles published in peer reviewed scientific journals; articles concerning the children’s own family companion animals, and where the maximum age of the participants was 18. A diagnosis of developmental disorders (such as autism spectrum disorder) or other psychiatric diagnoses in study participants was an exclusion criterion.

Data was extracted according to a predefined coding protocol. The protocol included information regarding the child’s psychosocial health, the type of companion animal, participant gender and age, family composition, animal characteristics, duration and strength of the bond with the companion animal. Also instruments used to measure companion animal attachment or bond, was obtained. Further, we extracted information concerning the instruments used to measure, children’s attachment to their parents and bond with siblings. Additional data, such as publication date, first author, country, and type of study design, was extracted for study identification and exploratory purposes. All extracted data was listed in a Microsoft Excel sheet to conduct a narrative synthesis.

## 3. Results

### 3.1. Search results

**Figure 1:**
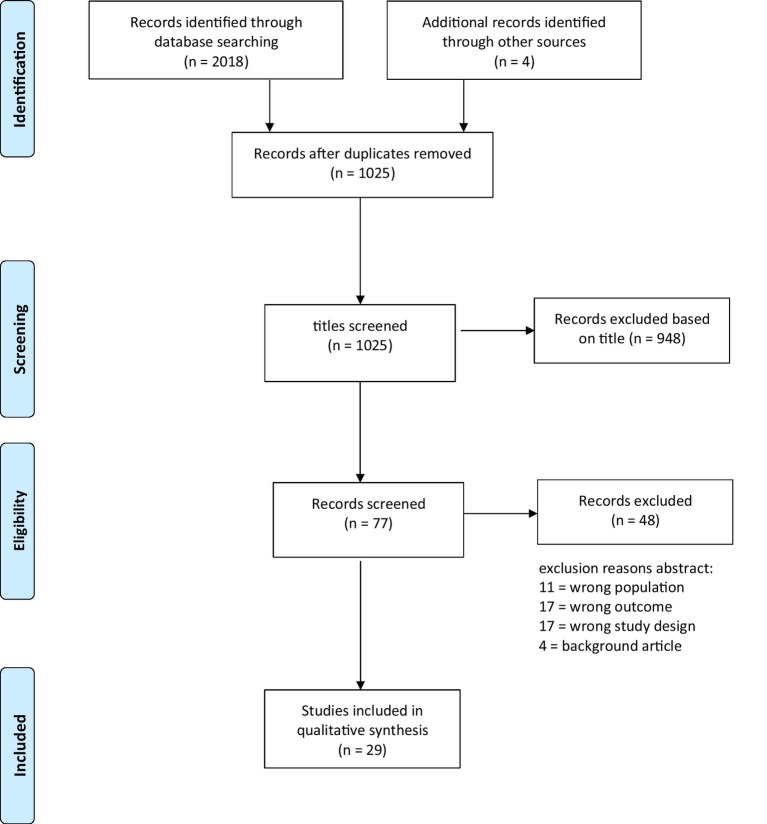
PRISMA flow diagram.

The initial search generated 2018 results (Web of Science *n* = 690; PubMed *n* = 144, APA PsycArticles: APA PsycInfo, Psychology and Behavioral Sciences Collection *n* = 1,184). Additionally, four records were obtained by screening the references of relevant articles. After removing duplicate records (*n* = 993), the titles of 1,025 articles were screened, of which 948 were excluded due to obvious inappropriate studies (i.e., PET scan, animals in Animal Assisted Interventions). 77 Publications were screened by the first and fifth author. Forty-eight articles were excluded for reasons such as an incorrect outcome or inappropriate study population. The final number of studies included in this systematic review was 29 (Figure 1).

### 3.2. Study characteristics

#### 3.2.1. Publication period and geographical origin

Publication dates ranged from 1990 to 2021, with the majority of articles published in 2017 (*n* = 7, Table 1). Almost two-thirds of the studies were published in the last decade. All but one studies had observational designs. In general, most studies had a cross-sectional quantitative survey design (*n* = 26), of which four where one time point of a longitudinal study. Three studies were longitudinal: one publications had a quantitative prospective cohort design, one had a quantitative panel survey design, and one study had a longitudinal qualitative philosophical design.

**Table 1:** Characteristics of the included studies.

	Author (year)	Country	Study design	Number (*n*) of participants	Participants age	Participants gender girls/boys	Companion animals	Pet attachment or bond instruments	Psycho-social health instruments
1	Black (2012)	United States	Cross-sectional study	293 of which 243 have pet(s)	13–18, mean 15.9 years	158/135	Dogs, cats, horses	CABS	RULS SSQSR
2	Bodsworth and Coleman (2001)	Australia	Cross-sectional study	80 (two parents) 61 (single parents)	3–12	74/67	Dogs	CABS, modified	
3	Carr and Rockett, 2017	United Kingdom	Longitudinal qualitative study	8	10–16	5/3	Dogs	Semi-structured interview, diagrammatic representations, guided diary for 6 months	
4	Cassels et al. (2017)	United Kingdom	Cross-sectional study, a wave of longitudinal study	31	12, mean 12.14 years (SD = 0.2)	12/9	Dogs, cats, rabbits, guinea pigs, hamsters, fish, chicken and unspecified	CENSHARE PAS-parent (mother’s perception of child pet bond)	NRI five field map
5	Daly and Morton (2003)	Canada	Cross-sectional study	136 77.4% with pets	9–14	75/61	Dogs, cats, fish, birds, reptiles other	CABS	Pet owner survey, Bryant index of empathy questionnaire, measure of emotional empathy, pet preference inventory
6	Daly and Morton, 2006	Canada	Cross-sectional study	155 (128 Bryant Empathy Index) 62% with pets	8–14		Dogs, fish, cats, rodents, reptiles, birds	LAPS	Pet owner survey, Bryant index of empathy pet preference inventory, PAS
7	Endenburg et al. (2014)	Netherlands	Longitudinal study t1 = 1992 t2 = 1994 t3 = 1997	451	3, 8 and 13 (t1)	248/203	Dogs, cats	CABS, modified	
8	Hall et al. (2016)	United States	Cross-sectional study	99	7–12, mean 10.25 (SD = 1.31)	49/50	Dogs	LAPS	modified NRI, SS, structured sociability assessment behavior coding: gazing and petting gesture
9	Hartwig and Signal (2020)	Australia	Cross-sectional study	286	14–19, mean 16.1 (SD = 1.3)	240/36 7 other	Dogs, cats, other	CABS	SSQ6, UCLA loneliness scale version 3
10	Hawkins and Williams (2017)	United Kingdom	Cross-sectional study	1,217 67% with pets	10–12, mean 9.7 (SD = 1)	620/597	Dogs, cats, small mammals, fish, reptiles, amphibians, birds, other	SAPS	FAS, PAS, CCA, CRHBA
11	Hirschenhauser et al. (2017)	Austria	Cross-sectional study	72 (group 1) 84 (group 2)	group 1 mean 8.5 (SD = 1.2) group 2 mean 11.4 (SD = 1.0)	group 1 38/34 group 2 44/40	Dogs	IPPA, (modified to pet attachment) 19 items from the RQ, modified to pet attachment *.	
12	Hoffman et al. (2013)	United States	Cross-sectional study, a wave of longitudinal study	92	11–18, mean 14.1 (SD = 1.7)	51/41	Dogs, owned for 4.7 years (sd = 2.9)	CENSHARE PAS	C-BARQ, modified PAS, DCRI
13	Jarolmen (1998)	United States	Cross-sectional study	163	6–17	95/68	Dogs, cats, birds, guinea pigs, other	CENSHARE PAS	Grief experience inventory, length of pet’s illness
14	Kerns et al. (2017)	United States	Cross-sectional study	99	9–11, mean 10.63	51/48	Dogs	Pet provision of support (4 items)	SS friendship quality questionnaire child behavior with dog – observational physical contact (petting, hugging), talking to the dog child anxiety related emotional disorders teacher-child rating scale
15	Kidd and Kidd (1990)	United States	Cross-sectional study	700		361/339		Melson parent questionnaire (demographic data, information on pet ownership, species, activities with pet, interest in pet and responsibility for pet)	Wilson pet attitude inventory for pet owners
16	Linder et al. (2017)	United States	Cross-sectional study	group 1 (12 overweight/obese) group 2 (31 healthy weights)	Group 1 = 8–12, mean 10.5 Group 2 = 8–13, mean 11	Group 1 11/12 Group 2 20/11	Dogs	Pet relationship scale, modified	Child and adolescent social support scale
17	Marsa-Sambola et al. (2017)	United Kingdom	Cross-sectional study	2,262	11–15, girls mean 13.5 (SD = 1.6) boys mean 13.02 (SD = 1.5)	1221/1041	Dogs, cats	SAPS	Items about health behavior in school children KIDSCREEN 10 index (quality of life)
18	Melson et al. (1991)	United States	Cross-sectional study	120	Kindergarten, second-grade, and fifth-grade	60/60	Dogs, cats	Behavioral attachment: items about various pet related activities. Affective attachment: a parent report of the child’s attachment to pet and an adapted CABS. cognitive attachment: open ended items regarding the child’s own pet and dogs and cats in general.	Bryant index of empathy for children and adolescents the pictorial scale of perceived competence and social acceptance for young children
19	Mueller and Callina (2014)	United States	Cross-sectional study	286 (military families) 70.8% children with pet(s)	mean 15.0	172/14	Dogs, cats, fish, reptiles, small rodents, chickens, rabbits, birds, horses, other species	CABS	Positive youth development: competence, confidence, connection, character and caring, CESD, 11-item perceived stress scale, A-COPE
20	Mueller et al. (2021)	United States	Cross-sectional study, 2 waves of longitudinal study	318	mean at t1 12.40 (SD = 1.07) mean at t2 13.00 (SD = 1.04)	207/108	Dogs and other pets	NRI-pet	Loneliness with a three-item scale. Coping with stress questions
21	Muldoon et al. (2019a)	United Kingdom	Cross-sectional study, a wave of longitudinal study	2,472	11, 13 and 15	1280/1191	Dogs, cats and small mammals	SAPS	
22	Muldoon et al., 2019b	United Kingdom	Cross-sectional study	6,700 age 11 (1,021 boys, 1,044 girls) age 13 (1,060 boys, 1,043 girls) age 15 (1,209 boys, 1,323 girls)	11, 13, and 15	3410/3290	Dogs, other	SAPS	Kid screen 10 index health and well-being, GHQ-12, single items from the HBSC
23	Paul and Serpell (1996)	United States	Cross-sectional study prospective, (12 months)	56 (27 dog’s owners, 29 non-dog owners)	8–12		Dogs	Visual analogue scale (mothers rated the child)	Questionnaires to monitor changes in the lives of middle childhood and their families during the first year of a new pet dog
24	Poresky (1996)	United States	Cross-sectional study	88 50% with pets, 50% without pets	3–6, mean 4.3 (SD = 1.0)		Dogs, cats	CABS	The Iowa social competency scales, the young children empathy measure
25	Poresky (1996)	United States	Cross-sectional study	88 50% with pets, 50% without pets	3–6, mean 4.3 (SD = 1.0)		Dogs, cats	CABS	Parental home assessment index, the Iowa social competency scales, the Denver prescreening developmental questionnaire, the young children empathy measure, Peabody picture vocabulary test
26	Lookabaugh Triebenbacher (1998)	United States	Cross-sectional study	174 (122 with a pet)	3–11	94/80	Dogs, cats, other	Structured interview with questions derived from the CABS, Pet attitude inventory and the Pet attitude scale.	
27	Van Houtte and Jarvis (1995)	United States	Cross-sectional study	130	8–13	59/71		Attachment to animals’ questions developed by Stallones et al. (1988)	Autonomy measure (Steinberg and Silverberg), self-concept scale for children, Rosenberg self-esteem scale
28	Vidovic et al. (1999)	Croatia	Cross-sectional study	826 (450 with pets; 376 without pets)	9–14	425/401	Dogs, cats, other	CPAS	Child empathy scale, child prosocial orientation scale, child loneliness scale, social anxiety scale for children, perception of family climate scale
29	Westgarth et al. (2013)	United Kingdom	Cross-sectional study	601 (381 completed PAS)	9–10		Dogs, cats, rodents, rabbits, horses, fish, other	CENSHARE PAS	

t1, t2 and t3, time points; SD, standard deviation; *, the only instruments that measure companion animal attachment styles; CABS, Companion animal bonding scale; CENSHARE PAS, Center for the Study of Human–Animal Relationships and Environments – Pet attachment survey; LAPS, Lexington attachment to pets scale; IPPA, Inventory for parent and peer attachment; RQ, Relationship Questionnaire; NRI, Network of relationships inventory; SAPS, Short attachment to pets scale; CPAS, Child pet attachment scale; RULS, Revised UCLA loneliness scale; SSQSR, Social support questionnaire, short form; PAS, Pet attitude scale; SS, kerns security scale; SSQ6, Short social support questionnaire; FAS, Family affluence scale; CCA, compassion to animals for children; CRHBA, Children’s reported humane behavior toward animals; C-BARQ, Canine behavioral assessment and research questionnaire; DCRI, dog care responsibility inventory; CESD, the center for epidemiological studies depression; A-COPE, adolescent coping orientation for problem experiences; GHQ-12, The general health questionnaire; HBSC, The Health Behavior in School-aged Children.

The majority of the first authors are from the United States of America (USA) (*n* = 15), followed by the United Kingdom (UK) (*n* = 7), Australia (*n* = 2), and Canada (*n* = 2). Three of the articles were published by authors from non-English speaking countries namely, Austria, Croatia, and Netherlands.

#### 3.2.2. Participants

The youngest participants in the included studies were 6 months old, with the most prevalent age group being 8 and 12 years old (Table 1). One study (Melson et al., 1991) did not specify age, but rather school classes (kindergarten to fifth-grade). In one study, the participant’s age ranged from 6 months to 18 years (Kidd and Kidd, 1990). Nine studies collected data regarding the family composition (Kidd and Kidd, 1990; Melson et al., 1991; Van Houtte and Jarvis, 1995; Paul and Serpell, 1996; Bodsworth and Coleman, 2001; Black, 2012; Westgarth et al., 2013; Carr and Rockett, 2017; Hartwig and Signal, 2020); however, this information was not always considered as a variable in the analyses. The number of participants varied greatly between studies from eight in a qualitative study (Carr and Rockett, 2017) to 6,700 in a quantitative study (Muldoon et al., 2019b). Nine articles had between 8 and 99 participants, 11 articles included 100 to 299 participants, and nine articles had over 300 participants. Five articles did not record the participant gender; however, the remaining articles had a comparable number of boys and girls. Participant ethnicity was disclosed by eight articles (Kidd and Kidd, 1990; Van Houtte and Jarvis, 1995; Triebenbacher, 1998; Black, 2012; Hoffman et al., 2013; Westgarth et al., 2013; Kerns et al., 2017; Mueller et al., 2021); however, only Westgarth et al. (2013) included this variable in their statistical analyses. Family socio-economic background was described by five articles (Melson et al., 1991; Van Houtte and Jarvis, 1995; Paul and Serpell, 1996; Hawkins and Williams, 2017; Linder et al., 2017), and the number of siblings was recorded by five articles (Melson et al., 1991; Van Houtte and Jarvis, 1995; Paul and Serpell, 1996; Westgarth et al., 2013; Hirschenhauser et al., 2017). The geographic location of the participants was described by three articles (Paul and Serpell, 1996; Bodsworth and Coleman, 2001; Hartwig and Signal, 2020). Perception of family climate (Vidovic et al., 1999) and the weight of the children (Body Mass Index) have both been described once (Linder et al., 2017). One article included only military families (Mueller and Callina, 2014). One article mentioned the quality of the home environment described by the parents (Poresky, 1996). Three articles described the preferred companion animal type by children (Daly and Morton, 2003, 2006; Mueller and Callina, 2014) and nine articles included participants with and without companion animals (Poresky and Hendrix, 1990; Van Houtte and Jarvis, 1995; Paul and Serpell, 1996; Poresky, 1996; Vidovic et al., 1999; Daly and Morton, 2003, 2006; Mueller and Callina, 2014; Hawkins and Williams, 2017).

#### 3.2.3. Companion animals

Of the 29 included studies, two articles did not define the animals used as companions (Kidd and Kidd, 1990; Van Houtte and Jarvis, 1995). The remaining studies reported dogs as the participants’ companion animals, of which seven studies included only dogs, five studies included dogs and cats, and 15 studies included dogs, cats, and other animals such as small mammals, rabbits, rodents, horses, reptiles, birds, and fish (Table 1).

Two studies mentioned the duration of the bond with the family dog, which was defined by the length of time of owning the dog (Bodsworth and Coleman, 2001; Hoffman et al., 2013). In the study by Hoffman et al. (2013), the family owned the dog for about 4.7 years, (SD = 2.9 years). The study by Bodsworth and Coleman (2001) did not describe the exact duration of the bond. Three studies described the duration of the bond with multiple animals (Black, 2012; Westgarth et al., 2013; Hirschenhauser et al., 2017). Only the study by Westgarth et al. (2013) included a table with the time the companion animal was owned (<1 year, 1–5 years, >5 years, all their lives). In a study regarding children in foster-care, the time the companion animal had spent in their current family was reported (7–13 months) and was, therefore, interpreted as the duration of the bond (Carr and Rockett, 2017).

The number of companion animals in the household at a given time was described by four articles (Kidd and Kidd, 1990; Melson et al., 1991; Black, 2012; Hawkins and Williams, 2017). Dog behavioral characteristics (Hoffman et al., 2013), the age of the dog (Linder et al., 2017), and the average daily time spent with the companion animal (Mueller et al., 2021) were mentioned once.

#### 3.2.4. Psychosocial health measures

The included articles used a variety of instruments to measure psychosocial health outcomes such as empathy, autonomy, family climate, self-esteem, depression, stress, coping, social network, social competence, and loneliness (Table 1).

### 3.3. Correlations between child-companion animal attachment/bond and psychosocial health

#### 3.3.1. Social support and social competence

A wide variety of instruments were used to measure the psychosocial health of the participants (see Table 1 for the overview and abbreviations of the instruments). Two articles described the relationship between the child-companion animal bond, the number of humans within the social network and the overall satisfaction with this network (Black, 2012; Marsa-Sambola et al., 2017). Black (2012) found that the strength of the bond with companion animals, measured with the CABS, was positively correlated to the number of humans in the social support network. A bigger social network was related to a stronger bond with companion animals. Further, a moderately negative correlation was observed between the strength of the companion animal bond and the feeling of loneliness; a stronger bond meant feeling less lonely. Moreover, children with more companion animals in their social network were less satisfied with the social support from humans compared to children with a few or no companion animals. Nevertheless, Marsa-Sambola et al. (2017) found that a stronger bond with dogs also meant better communication between the children and their significant others such as their mother, father and best friend.

Studies investigating the social development of children and the strength of the companion animal bond revealed inconclusive results. A positive correlation was found between the strength of the companion animal bond and being cooperative, outgoing and accepting of others (Poresky and Hendrix, 1990; Poresky, 1996). Whereas in the study by Kerns et al. (2017), affection for the family dog and the experience of companionship were not significantly related to social–emotional competence. Also, Van Houtte and Jarvis (1995) found no significant correlation between the strength of the companion animal bond and autonomy, self-concept and self-esteem in children. Further, the study by Linder et al. (2017) found that obese/overweight children who perceived lower social support by peers, described a stronger bond with dogs, compared to healthy weight children. However, the authors did not find a correlation between the strength of the bond with dogs and the total perceived social support by peers.

#### 3.3.2. Empathy

Some studies examined the relationship between the strength of the companion animal bond and children’s empathy (Poresky and Hendrix, 1990; Melson et al., 1991; Vidovic et al., 1999; Daly and Morton, 2003, 2006). Overall, a positive association was found between the strength of the companion animal bond and a child’s empathy, however, this was affected by age and gender. In kindergarten and fifth grade children, a positive relationship between the strength of the child-companion animal bond and empathy was found (Melson et al., 1991; Poresky, 1996), whereas a negative correlation between the strength of the child-companion animal bond and empathy in boys in second grade was observed (Melson et al., 1991). Daly and Morton (2003) did not observe a relationship between empathy and the strength of the bond with companion animals, measured with the CABS. Nevertheless, with the use of the LAPS, Daly and Morton (2006) found a relationship between the strength of the bond and empathy. This effect was weaker in children who only had a cat, compared to children who had both cats and dogs.

#### 3.3.3. Quality of life, and coping with stress and anxiety

The study by Marsa-Sambola et al. found that a stronger bond with dogs was associated with a better quality of life (Marsa-Sambola et al., 2017). Also, a higher pet bonding score was associated with more adaptive coping strategies such as prosocial orientation (Vidovic et al., 1999). Moreover, proactive orientation and participating in social activities in children with a military deployed family member (which is assumed to be associated with higher stress due to separation from family members, increased risk of deployment and frequent moves throughout the country) were also associated with a strong companion animal bond (Mueller and Callina, 2014).

Further, Kerns et al. (2017) found no correlation between children’s anxiety and the bond with their dog.

### 3.4. Correlations between the strength of the child-companion animal bond and children’s and companion animal’s characteristics

#### 3.4.1. Companion animal species

Twenty studies included a diversity of companion animals such as dogs, cats, small mammals, horses, reptiles, birds, and fish. The strength of the child-companion animal bond was associated with animal species. Eighteen studies described that the bond between children and dogs was the strongest, followed by cats, horses, small mammals (rodents, mice, rabbits), reptiles, birds, and then fish. However, Melson et al. (1991) investigated the bond between young children with dogs and cats and found no significant difference between the children’s attachment related affections (talking about companion animal, shows affection, ignores companion animal) and cognitions (feelings about companion animal and knowledge of characteristics and care of companion animal), to dogs and to cats. Also, the study by Hirschenhauser et al. (2017) found no correlation between the attachment quality measured with IPPA and companion animal species.

#### 3.4.2. Dog characteristics

Three studies described the specific characteristics of dogs associated with the strength of the bond (Hoffman et al., 2013; Hall et al., 2016; Kerns et al., 2017). The experimental study by Hall et al. (2016) involving 99 children aged 7–12 years, and their family dog. They assessed the strength of the bond with the LAPS, the perceived support (such as disclosing your thoughts, receiving help) of the dog with a dog-modified NRI and attachment security to their parent. An observation was conducted during two experiments. In the first experiment children had to sit quietly in a room, call the dog, and were only allowed to interact when the dog approached within 1 m. In the second experiment the response of the dog was observed when the child pointed to one of two objects (cans), to see if the dogs followed the nonverbal command. Behaviors such as gazing, petting and the dog following gestures of the child were obtained. The authors found that children form stronger bonds with dogs who are more supportive. Also, a higher LAPS score was obtained with dogs who listened to commands and when a less spontaneous interaction during the first experiment was observed between the child and the dog.

The studies by Hall et al. (2016) and Hoffman et al. (2013) found that being responsible for the dog and taking care of the dog was associated with the development of a stronger bond, as was the trainability of the dog. Also, Kerns et al. (2017) showed that the time the children spend petting the dog contributed to the development of a stronger bond.

#### 3.4.3. Duration of the bond between children and their companion animals

It remains unknown whether the strength of the bond between children and their companion animals is associated with the time the children have had with their companion animals. Although five studies collected data on the duration of the bond, only three studies estimated a correlation between the strength of the bond and the duration (Bodsworth and Coleman, 2001; Endenburg et al., 2014; Mueller et al., 2021). Bodsworth and Coleman (2001) found that the duration of the bond was negatively correlated to the strength of the bond between the child and companion animal. However, Endenburg et al. (2014) found a positive correlation between the duration and the strength of the bond between children and companion animals. In the latter study, the strength of the bond with dogs increased for children who had the same dog for 5 years. Further, Mueller et al. (2021) found stability in a period of 6 months. The two longitudinal studies by Endenburg et al. (2014) and Mueller et al. (2021) indicate that stability or a stronger bond evolves with an increase in the duration of the time spent with companion animals.

#### 3.4.4. Children’s age

Ten articles studied the relationship between the strength of the companion animal bond and the age of the children (Melson et al., 1991; Jarolmen, 1998; Vidovic et al., 1999; Black, 2012; Endenburg et al., 2014; Hawkins and Williams, 2017; Hirschenhauser et al., 2017; Muldoon et al., 2019a,b; Mueller et al., 2021). The results are incongruent, as three studies found a positive relationship with age (Melson et al., 1991; Endenburg et al., 2014; Hirschenhauser et al., 2017), three studies found a negative relationship with age (Vidovic et al., 1999; Muldoon et al., 2019a,b), and four studies found no correlation between age and the strength of the companion animal bond (Jarolmen, 1998; Black, 2012; Hawkins and Williams, 2017; Mueller et al., 2021). Only Hirschenhauser et al. (2017) studied the correlation between companion animal attachment and children’s age. With the IPPA (adapted to companion animals), they found that attachment quality to companion animals did not differ between children aged 6–10 and 11–14.

#### 3.4.5. Children’s gender

Thirteen articles described the relationship between children’s gender and the strength of the companion animal bond (Melson et al., 1991; Vidovic et al., 1999; Black, 2012; Westgarth et al., 2013; Endenburg et al., 2014; Hall et al., 2016; Hawkins and Williams, 2017; Hirschenhauser et al., 2017; Marsa-Sambola et al., 2017; Muldoon et al., 2019a,b; Hartwig and Signal, 2020; Mueller et al., 2021). Nine studies found that the scores on companion animal bonding scales for girls was higher than those for boys, indicating that girls tend to form stronger bonds (Melson et al., 1991; Vidovic et al., 1999; Black, 2012; Endenburg et al., 2014; Hawkins and Williams, 2017; Hirschenhauser et al., 2017; Marsa-Sambola et al., 2017; Muldoon et al., 2019a,b). In the study by Hirschenhauser et al. (2017) girls and boys aged 6–10 reported no difference, but girls in the age group 11–14 did report a stronger bond. Four articles did not observe any significant difference in the strength of the companion animal bond with regards to gender (Westgarth et al., 2013; Hall et al., 2016; Hartwig and Signal, 2020; Mueller et al., 2021). Only one study reported a stronger bond to companion animals by boys in kindergarten when compared to girls of the same age (Melson et al., 1991). However, this study found no gender difference in the strength of the bond for children aged 6–11. Moreover, in the group of children older than 11 years, girls described a stronger bond with companion animals than boys.

#### 3.4.6. Family characteristics

Three of the included 29 articles described the relationship between several family characteristics and the strength of the child-companion animal bond. Bodsworth and Coleman (2001) reported a negative correlation between family income and the strength of the child-companion animal bond. However, in the studies by Hawkins and Williams (2017) and Westgarth et al. (2013), there was no significant correlation between family affluence scores and the strength of the child-companion animal bond. Bodsworth and Coleman (2001) found that children from single-parent families showed higher levels of bonding with dogs compared to children from two-parent families. The number of siblings was not related to the strength of the companion animal bond. In the study by Westgarth et al. (2013), single children reported a stronger bond to their favorite companion animal compared to children who had siblings. Cassels et al., 2017 found no significant correlation between the companion animal bond and sibling relationships measured with the NRI scales disclosure and companionship. Nevertheless, the authors stated that the children were more satisfied with their companion animal bond than with their sibling bond, and they experienced less conflicts with their companion animals than with their siblings. Ethnicity (white United Kingdom, black United Kingdom, Pakistani, Indian, Bangladeshi, Chinese, Somali, mixed or other), was not associated with the strength of the companion animal bond in the study by Westgarth et al. (2013).

### 3.5. The correlation between the child-companion animal bond, attachment to parents and the relationship with siblings

One study compared the quality of the relationships between siblings and companion animals (Cassels et al., 2017). They found that children reported greater satisfaction and less conflict with companion animals compared to siblings, but no difference on experiencing companionship and disclosre. They discuss that companion animal and sibling relationships seem to be characterized by some similar distinct dimensions. They state that the companion animal bond could have the same effect on socio-emotional development as sibling relationships. Two articles studied the association between attachment to parents and the bond with dogs (Hall et al., 2016; Kerns et al., 2017). Both studies applied the Kerns Security Scale, an instrument intended to measure the construct of attachment security. These studies indicated that a more secure attachment style to parents is positively related to a stronger bond with dogs. The study by Kerns et al. (2017) showed that secure mother–child attachment, but not father-child attachment, predicted more physical interactions of the children with the family dog, which is correlated with a stronger bond with the dog. Further, a secure attachment to both mother and father was significantly related to the child’s affection toward the dog, companionship, and admiration of the dog. Hall et al. (2016) showed that a secure attachment to parents was mildly correlated (*β* = 0.15), with a stronger bond with the family dog.

The study by Hirschenhauser et al. (2017) was the only one that measured attachment styles with a companion animal adapted RQ and IPPA.They found that 94% (*n* = 146) of their participants had a secure attachment style to companion animals, while 2% had a preoccupied, 3% a dismissing and 1% a fearful attachment style. This could indicate that child-companion animal attachment is different from child–parent attachment since the general prevalence of attachment style to humans is inconsistent with these results.

Carr and Rockett (2017) published a qualitative study on attachment between foster children, companion animal dogs and their foster parents. They investigated whether the bond with the family dog reflects features of a secure attachment, and if children’s attachment to the family dog could facilitate a secure attachment with foster caregivers. For that, eight foster children, age 10–16 years, were followed for 6 months. Seven out of 8 children described the dogs as a safe haven, and five children saw the dog as a secure base. They felt more confident with the dog around and protected when the dog slept nearby. The interaction of the foster caregivers with the family dog was associated with how the children viewed the foster caregivers. When the children saw the foster caregivers as kind and caring to the dog, they were able to trust them more. Carr and Rockett (2017) discussed the idea of the human-animal bond as a relationship facilitator, who “offer a pathway toward (re)establishing attachment security in the context of human attachments.”

### 3.6. Instruments used to measure the child-companion animal bond

The studies used a variety of scales or questions to measure the children’s bond with companion animals (Table 1, but also see Anderson (2007) for a complete overview of existing instruments on the human-animal bond). The Companion Animal Bonding Scale (CABS) was the most commonly used measure (*n* = 8), followed by the Pet Attachment Survey (CENSHARE PAS, *n* = 4). Three studies used the Short Attachment to Pets Scale (SAPS) and two studies used the Lexington Attachment to Pets Scale (LAPS). The following instruments were mentioned only once: the Pet Relationship Questionnaire (modified), the Relationship Questionnaire (RQ; modified), the Pet Attachment Survey, a Visual Analogue Scale (mother’s rating), the Melson Parent Questionnaire, the Pet Provision of Support, Network of Relationships Inventory-Pet (NRI-Pet), and Inventory for Parent and Peer Attachment (IPPA, modified for companion animal attachment). One study used their own developed questions about attachment to animals (Van Houtte and Jarvis, 1995). Melson et al. (1991) developed items about various companion animal-related activities that were interpreted as behavioral attachment. Affective attachment was determined with a newly developed scale with questions derived from the CABS. Cognitive attachment was determined with open ended items describing feelings about their own companion animal, and knowledge about characteristics and care of dogs and cats in general. In this study, they found that the three attachment dimensions were moderately related, even when the items were answered by both the child and parent (Melson et al., 1991).

A qualitative study developed a semi-structured interview and used diagrammatic representations of the foster parent and dog to discuss the relationships in relation to each other. The participants also maintained a guided diary for 6 months in which they recorded prominent events, and their feelings and thoughts about these events (Carr and Rockett, 2017). Another study developed a structured interview, with questions derived from the CABS, the Pet Attitude Inventory and the Pet Attitude Scale (Triebenbacher, 1998).

We identified one study that used a modified instrument on human-human attachment to determine child-companion animal attachment style, the RQ and IPPA (Hirschenhauser et al., 2017). All other included child – companion animal bond instruments, besides the RQ and IPPA, measure the intensity of the bond and some attachment components, but they do not identify the different attachment styles such as secure, avoidant, and anxious. The IPPA and the RQ are conceptualized as dimensions of attachment anxiety and avoidance (adapted to companion animal attachment). These instruments describe attachment to a companion animal as a secure or insecure (anxious or avoidant) attachment style.

## 4. Discussion

This systematic review aimed to summarize and evaluate current knowledge regarding children’s bonds with companion animals and their psychosocial health outcomes. Further, we described the characteristics of children and companion animals associated with a strong child-companion animal bond. We also described correlations between attachment to parents, the relationship with siblings and the child-companion animal bond. Finally, we presented an overview of the instruments currently used to measure the child-companion animal bond and attachment.

A total of 29 studies were included in this review. Empathy, quality of life and adequate coping are positively correlated to the strength of the child-companion animal bond. However, the results were inconclusive for social development, social support and social competency. Further, one study found that the strength of the child-companion animal bond is not associated with children’s self-esteem, self-concept, autonomy, or anxiety. Since most studies had a cross-sectional design, and results were inconclusive, it is indicated that more research is needed on this subject. Also, due to the diversity of measures, it is difficult to generalize the findings. We found that the strength of the child-companion animal bond was associated with animal species, as children seem to form the strongest bond with dogs, followed by cats and other mammals. The children’s gender was also associated with the strength of the child-companion animal bond, as girls often reported a stronger bond with companion animals. These results seem to be consistent with findings regarding an adult’s bond with companion animals, in which females also reported a stronger bond compared to males (Herzog, 2007; Smolkovic et al., 2012; Martens et al., 2016; Meehan et al., 2017; Testoni et al., 2017).

It remains unclear whether the duration of the bond between children and their companion animal is associated with the strength of the bond. Surprisingly, only three out of 29 studies described a correlation between the duration and the strength of the bond, and the results were inconclusive. Not every bond with a companion animal develops into an attachment. For instance, the bond with companion animals that have a short life expectancy might be prone to being companionship rather than an attachment, even though they can still fulfill some of the children’s attachment needs. Studies show that the duration of the human-animal bond is associated with less doctor visits and therefore could be an important factor in explaining the effects of the human-animal bond on wellbeing (Headey and Brabka, 2007; Headey and Grabka, 2007). We also found conflicting results for the association between children’s age and the strength of the child-companion animal bond. In addition, the association between family composition and the strength of the child-companion animal bond remains unclear.

Another goal of this study was to review the correlation between a child’s attachment to their parents and the child-companion animal bond. Although only three studies in this review investigated this aspect, it appeared as though a secure attachment to parents is associated with a stronger bond with companion animals, as well as with more affection, more companionship and a higher admiration for the family dog. Further, children in foster care regard a dog as a safe haven and secure base. It is suggested that the interaction of the foster caregiver with the dog is associated with the transfer of children’s attachment from the dog to the foster caregiver.

An aspect that could affect child-companion animal attachment is a changing human social network. The development of a bond or attachment must be seen in the context of a network of attachment figures and other important individuals (Thompson, 2021). The size and quality of the network of attachment figures could influence the children’s bond with their companion animals, but further research is required to establish this. For instance, children with a more insecure or disorganized attachment style to their parents could potentially have a more secure attachment style to companion animals (Julius et al., 2012). If this is true, the effect it might have on the children’s psychosocial health and development could inform us about the importance of the child’s attachment to companion animals. It remains unknown whether or not attachment to humans is transferrable to companion animals, and how attachment to companion animals is formed within this network. Therefore, a growing and changing network of attachment figures could influence attachment styles to companion animals. Even though the bond with a companion animal can be temporary, that does not mean that it is not important. Within animal assisted interventions, the relationship that patients form with the animals in therapy is often described as an attachment (Parish-Plass, 2008). Beetz et al. (2011) found that boys with an insecure/disorganized parental attachment style, who were exposed to a social stressor, experienced less stress in the presence of a dog, compared to a toy dog or a human. This could imply that the children’s insecure or disorganized attachment style to humans was not transmitted to companion animals, and also explain mechanisms in animal assisted therapy. Yet, further research on the transference of human-attachment to companion animal attachment is required.

Some of the contradictory results found in this review might be explained by the variety of instruments used to measure the child-companion animal bond. Ten out of twelve of the included instruments operationalized the strength of the child-companion animal bond on a behavioral, cognitive or emotional level. Although these levels are related to attachment components such as separation distress, secure base, safe haven or maintaining proximity, the instruments do not measure attachment styles. Further, only the SAPS was validated with children during construction of the questionnaire. The CABS questionnaire was used the most, namely in eight out of the 29 studies. The LAPS was used in two, and the SAPS in three studies. The LAPS and SAPS both adopted items from the CABS. The CABS is a behavioral self-report scale with a focus on the quality of the child-companion animal bond, describing the extent of child-companion animal activities. However, questions on the CABS about traveling with a companion animal, holding a companion animal, and a companion animal being responsive might be more oriented to dogs than other companion animals (Endenburg et al., 2014). Endenburg et al. (2014) also found that the CABS failed to adequately measure the concept of child-companion animal bond when translated to Dutch. Therefore, the content validity of the CABS might be inefficient.

Further, Daly and Morton (2003, 2006) found different effects on empathy when using the CABS and the LAPS. These findings could imply that the content and construct validity of these instruments might be inefficient. Also, the instruments may not fit the theoretical model of attachment as described by Bowlby, therefore it may be more appropriate to adapt human attachment instruments, with which we can study attachment styles, to companion animals. Only two such modified instruments to measure child-companion animal attachment were found. The IPPA and RQ are both modified instruments that are able to measure attachment styles and quality. The RQ measures attachment on two dimensions resulting in four attachment styles: secure, dismissing, preoccupied and fearful. Using RQ, Hirschenhauser et al. (2017) found that 94% of the participants had a secure attachment style to companion animals, 2% preoccupied, 3% dismissing, and 1% fearful. They found no differences in the attachment styles between companion animal species in 156 participants. This could indicate that attachment to companion animals is the same across companion animal species, compared to the strength of this bond, which is the highest for dogs. Further, the high percentage of secure attachment to companion animals could indicate that child-companion animal attachment is different from child–parent attachment since the prevalence of secure attachment style to humans and companion animals are different. Approximately 65% have a secure attachment style, 21% an anxious attachment style, 14% ambivalent attachment and a small percentage a disorganized attachment style to parents (Van Ijzendoorn, 1992). Therefore, attachment to companion animals might be different to attachment to parents.

### 4.1. Limitations and future directions

A limitation of this review is that only peer reviewed articles present in databases were included. This was done to ensure efficiency; however grey research could be helpful to counterbalance possible publication bias. The presented results need to be interpreted carefully as there is no information regarding causality in the included studies about attachment to companion animals, determinants of the child-companion animal bond, and its association with psychosocial health, since most of the study designs were cross-sectional. Although we aimed to include studies concerning children’s attachment to companion animals, only one out of the 29 articles used an instrument measuring attachment congruent with the attachment theory and attachment styles. The remaining articles used instruments that measure some aspects of attachment but mainly measure the strength of the bond. Still, most of these articles used the terminology “attachment” even though the strength of the bond is not the same concept as an attachment.

With this article we contributed to the research on the human-animal bond as we provided a systematic review depicting the current knowledge regarding the association between a child’s bond with companion animals and their psychosocial health. We gathered information regarding the characteristics of children and companion animals and the strength of their bond, correlations between attachment to parents and the child-companion animal bond, and instruments that measure the child-companion animal bond. Based on these findings, we recommend that future research need to be clear in regard to the relationship they are investigating. It is important to clearly distinguish between attachment, ownership, companionship, social support, or an affiliative bond. We therefore suggest that future studies incorporate validated attachment instruments that are able to measure attachment style or quality to companion animals. In addition, other relevant aspects should be included within future studies, such as including instruments on emotion regulation strategies. Children with a secure attachment have more adaptive coping strategies, however we do not know if this also applies to a secure attachment to companion animals. This knowledge could help identify if or when children co-regulate their emotions with companion animals, and if this has a positive effect on children’s wellbeing. Also, including instruments that measure attachment to humans could help identify whether or not attachment styles are transferred from humans to companion animals. Finally, it is important to use longitudinal prospective studies and to use designs where a causal relationship between attachment to companion animals and psychosocial health can be studied.

## 5. Conclusion

The current evidence suggests a correlation between the strength of the child-companion animal bond and psychosocial health domains such as empathy, quality of life and adequate coping. No correlation was found with self-esteem, self-concept, autonomy or anxiety. However, these results must be interpreted with caution because the current research provides an inconsistent picture. The strength of the child-companion animal bond seems to be related to gender and animal species and possibly to having siblings. However, further research is needed on these subjects, since results regarding duration of the bond, age and family characteristics are inconclusive. Research also shows a moderate correlation between attachment to parents and the strength of children’s companion animal bond. Since we could only find two studies reporting this correlation, more research is required.The bond between children and their companion animals has attachment related features, yet the current instruments are not able to measure attachment style or quality, as most instruments are developed to measure the strength of the bond.

Because of inconclusive results, a diversity of used instruments and low quality of evidence, future research with a high level of evidence is needed to be able to measure children’s attachment styles to companion animals and correlations with psychosocial health and socio-emotional wellbeing. Whether or not attachment to companion animals contributes to children’s psychosocial health, and whether children’s attachment styles to humans and companion animals are comparable, remains unknown.

## Data availability statement

The raw data supporting the conclusions of this article will be made available by the authors, without undue reservation.

## Author contributions

DG created the framework of this review and performed the search and wrote the first draft of the manuscript. DG and TW selected the papers. KH and M-JE-S supervised the work. DG, RL, TW, AD, KH, and M-JE-S were involved in developing the protocol, conducting the study and the revision of the manuscript. All authors contributed to the article and approved the submitted version.

## Funding

KH receives funding from the Swiss National Science Foundation under an Eccellenza grant (no. PCEFP1_194591/1).

## Conflict of interest

The authors declare that the research was conducted in the absence of any commercial or financial relationships that could be construed as a potential conflict of interest.

## Publisher’s note

All claims expressed in this article are solely those of the authors and do not necessarily represent those of their affiliated organizations, or those of the publisher, the editors and the reviewers. Any product that may be evaluated in this article, or claim that may be made by its manufacturer, is not guaranteed or endorsed by the publisher.
